# Meeting Abstracts of the 1st Fragile X International Congress

**DOI:** 10.1186/s13023-025-03574-x

**Published:** 2025-04-24

Editors: Professor Gaia Scerif (1) and Dr Kirsten Johnson (2)

1. Professor of Developmental Cognitive Neuroscience, University of Oxford and St. Catherine's College, Department of Experimental Psychology, Anna Watts Building, OX2 6GG, Oxford, UK
2. President, Fragile X International, 11 Rue d’Egmont, 1000-Bruxelles, Belgium

The aim of this Congress was to bring together clinicians and researchers working in the area of Fragile X Syndrome (FXS) and Fragile X Premutation Associated Conditions (FXPAC) from around the world, giving them the opportunity to network, be informed of the latest research from colleagues, workshop ideas through panel discussions, discuss translational research with clinical applications, and form relationships of future collaboration.

The speakers’ presentations covered various FMR1 premutation issues, biomarkers, behavioural aspects, treatment of FXS, mosaicism, models of care and expert centres in various countries, and more. A recently published paper, ‘A Holistic Approach to Fragile X Syndrome’, was presented and provides the reader with an overview of the scope of the Congress. Prof Scerif's contribution was supported by ESRC UKRI grant ES/X013561/1.

## P1 Development of molecular biomarkers as susceptibility/risk of development, progression and severity of FXTAS

### Flora Tassone

#### Department of Biochemistry and Molecular Medicine, University of California Davis, School of Medicine, Sacramento, 95817 CA, USA. MIND Institute, University of California Davis Medical Center, Sacramento, 95817 CA, USA

*Orphanet Journal of Rare Diseases* 2025, **20(1)**: P1

**Background:** Carriers of the *FMR1* premutation (PM) are at increased risk for developing Fragile X-associated tremor/ataxia syndrome (FXTAS), a late-onset neurodegenerative disorder characterized by cerebellar gait ataxia, intentional tremor, neuropathy, and parkinsonism. Currently there are no treatments available to stop or slow down FXTAS progression and the only options available are focused on managing the symptoms. Thus, it is very important to identify a robust set of molecular biomarkers that can predict disease development, progression and severity, in addition, to facilitate clinical trials in FXTAS and predict efficacy of treatment response and be beneficial for the patients.

**Methodology:** Metabolomics and Proteomics analyses using mass spectrometry were carried out in male carriers of a PM with FXTAS, without FXTAS and compared to healthy controls without the premutation, with similar age and gender distributions.

**Results:** We quantitatively compared proteomics and metabolomics profiles in PBMCs/plasma of males with a PM and controls to identify biomarkers differentially expressed between the groups. Significant metabolic alterations in purine and sphingolipid metabolism were detected in participants with FXTAS, playing a potential contributive role in the development of the disease. We also demonstrate dysregulation of several protein pathways, particularly neurodegeneration-related proteins, in those involved in mitochondrial function and in those involved in neuroinflammation, all implicated in the pathogenesis of FXTAS. Importantly, a subset of the identified proteins, including those involved in mitochondrial function, were significantly associated with the CGG repeat number and correlates with FXTAS stage.

**Conclusion:** The significance in identifying molecular biomarkers and changes in the associated pathways, can objectively shed lights on the molecular mechanisms leading to disease pathology, and therefore be crucial for improving patient outcomes. Importantly, the identification of non-invasive blood-based biomarkers can provide insights for potential therapeutic targets that could lead to prevent or slow down symptoms of FXTAS.

## P2 Neuroimaging evidence of glymphatic system dysfunction in FXTAS

### Andrea Elias-Mas^1,2,3^, Cèlia Painous Martí^4^, Esther Granell Moreno^5^, Marta Rubio-Roy^2,6^, Jorge Aguado^7^, Emma Muñoz-Moreno^8^, Carlos Laredo^8^, César Garrido^8^, Sofia González Ortiz^9^, Randi Hagerman^10,11^, Jun Yi Wang^12^, Laia Rodríguez-Revenga^13,14^

#### ^1^Radiology Department, Hospital Universitari Mútua de Terrassa, Terrassa, Spain. ^2^Parc Taulí Research and Innovation Institute (I3PT), Sabadell, Spain. ^3^Genetics Doctorate Program, Universitat de Barcelona (UB), Spain.^4^Neurology Department, Hospital Clínic de Barcelona, Barcelona, Spain.^5^Radiology Department, Hospital de Santa Creu i Sant Pau, Barcelona, Spain.^6^Neurology Department, Parc Taulí Hospital Universitari, Sabadell, Spain.^7^Department of Child and Adolescent Psychiatry and Psychology, Institute of Neurosciences, Hospital Clinic Universitari, Institut d'Investigacions Biomèdiques August Pi i Sunyer (IDIBAPS), Barcelona.^8^Institut d'Investigacions Biomèdiques August Pi i Sunyer (IDIBAPS), Barcelona, Spain.^9^Radiology Department, Hospital Clínic de Barcelona, Barcelona, Spain.^10^MIND Institute, University of California Davis, Sacramento, CA, United States. ^11^Department of Pediatrics, University of California Davis Medical Center, Sacramento, CA, United States.^12^Center for Mind and Brain, University of California Davis, CA, United States.^13^Biochemistry and Molecular Genetics Department, Hospital Clinic of Barcelona-Institut d'Investigacions Biomèdiques August Pi i Sunyer (IDIBAPS), Barcelona, Spain.^14^CIBER of Rare Diseases (CIBERER), Instituto de Salud Carlos III, Madrid, Spain

*Orphanet Journal of Rare Diseases* 2025, **20(1)**: P2

**Background/objectives**: *FMR1* premutation carriers (55–200 CGG repeats) are at risk of developing fragile X-associated tremor/ataxia syndrome (FXTAS), a neurodegenerative disorder associated with motor and cognitive impairment. The glymphatic pathway is an important brain homeostatic mechanism allowing the clearance of interstitial fluid and solutes and its failure is a final common pathway to dementia. The aim of this exploratory study is to assess the activity of the glymphatic system and brain metabolomics in FXTAS using Magnetic Resonance Imaging (3T MRI).

**Methods:** Four carriers with FXTAS, five carriers without FXTAS and twelve controls underwent 3T MR scan (including diffusion-weighted imaging sequence and 1H-Magnetic resonance spectroscopy (MRS) at the level of the right middle cerebellar peduncle), neurocognitive and CGG repeat testing.

**Results:** MRS showed lower Glutathione/ Creatine in carriers vs. controls (p= 0.049), indicating oxidative stress in carriers and a trend towards lower N-acetyl-aspartate/ Creatine was found in carriers vs. controls (p= 0.062), suggesting decreased neuronal integrity in carriers. A trend towards lower DTI-ALPS index (mean both hemispheres, p=0.054) was observed in FXTAS vs. controls, potentially indicating glymphatic dysfunction in FXTAS.

**Conclusion:** Signs of oxidative stress and reduced neuronal integrity were found in the brains of FMR1 premutation carriers, and glymphatic dysfunction could potentially contribute to the development of FXTAS in FMR1 premutation carriers.

**Grants:** This study was supported by Instituto de Salud Carlos III (ISCIII), (through the project PI21/01085), co-funded by the European Union and SERAM-INDUSTRIA de investigación 2022.The CIBER de Enfermedades Raras is an initiative of the Instituto de Salud Carlos III.

## P3 Care for patients with FXTAS

### Dr Sundus Alusi

#### The Walton Centre NHS Foundation Trust, Liverpool, UK

*Orphanet Journal of Rare Diseases* 2025, **20(1)**: P3

Patients with Fragile X associated tremor and ataxia syndrome (FXTAS) typically present to neurology services with tremor or gait ataxia impairing their daily activities and resulting in disability. Problems with cognition and executive function may not be as obvious at presentation but when present, they can cause significant difficulties for patients and their families. Recognising the spectrum of the clinical deficits is crucial for effective management.

Clinical care for patients who are concerned about FXTAS starts by assessments to confirm or refute the diagnosis, followed by explaining the condition and management options. There is currently no treatment that can cure or halt the progression of FXTAS. Tremor can be treated conservatively or with medications in a similar manner to other tremulous conditions. The management of ataxia is aimed at the enablement of safe mobility and minimising the risk of falls and injury by providing adaptations to the surroundings and improving bone health. Genetic counselling and discussing other premutation related conditions is also included in the clinical care.

## P4 FMR4 as a potential blood biomarker for fragile X-associated primary ovarian insufficiency

### Laia Rodríguez-Revenga

#### Hospital Clinic Barcelona, Spain

*Orphanet Journal of Rare Diseases* 2025, **20(1)**: P4

**Background/objectives:** Female *FMR1* premutation carriers are at risk for developing fragile X-associated primary ovarian insufficiency (FXPOI), a condition characterized by amenorrhea before age 40 years. Not all *FMR1* premutation women suffer from FXPOI and nowadays there are no biomarkers that can predict the occurrence. Long non-coding RNAs (lncRNAs) comprise a group of regulatory transcripts. Previously, we described a significant association between FXPOI and high expression levels of FMR4 (FMR1-derived lncRNA), suggesting a potential role of FMR4 as a possible biomarker for FXPOI (Alvarez-Mora el al. 2022). A limitation in the study design was that it was exploratory. Herein, we further examined the role of FMR4 as biomarker to assess the risk of developing FXPOI, by characterizing young *FMR1* premutation female carriers who have not been diagnosed as FXPOI.

**Methods:** Serum anti-Müllerian hormone (AMH) level and antral follicle count (AFC) were used to assess woman’s ovarian reserve. FMR4 transcript level was evaluated in total RNA extracted from peripheral blood by digital droplet PCR.

**Results:** A negative association was found between AMH, AFC and FMR4 (R^2^ = 0.2 for AMH and R^2^ = 0.4 for AFC), suggesting that FMR4 might help as an additional marker predicting ovarian reserve.

**Conclusion:** FMR4 might help to better assess the risk of *FMR1* premutation women of developing FXPOI.

**Grants:** This study was supported by Fundación Merck Salud (19-FE-011) and Instituto de Salud Carlos III (ISCIII), (through the project PI21/01085), co-funded by the European Union. The CIBER de Enfermedades Raras is an initiative of the Instituto de Salud Carlos III.

## P5 Other FXPAC-related symptoms and conditions

### Dragana Protic^1,2^

#### ^1^Department of Pharmacology, Clinical Pharmacology and Toxicology, Faculty of Medicine, University of Belgrade, Belgrade, Serbia. ^2^Fragile X Clinic, Special Hospital for Cerebral Palsy and Developmental Neurology, 11000 Belgrade, Serbia

*Orphanet Journal of Rare Diseases* 2025, **20(1)**: P5

**Abstract** The premutation (PM) of the fragile X messenger ribonucleoprotein 1 (FMR1) gene is marked by an expansion of CGG trinucleotide repeats (55–200 CGGs) within the 5' untranslated region, along with elevated levels of FMR1 mRNA. Any health issues linked to this PM can be broadly classified as fragile X PM-associated conditions (FXPAC). In addition to the increased risk of psychiatric and mental health symptoms observed in carriers of PM alleles, there is also a higher prevalence of other FXPACs. For instance, previous research indicates that PM carriers are more susceptible to developing hypertension compared to the general population, possibly due to reduced or absent levels of FMRP. Additionally, metabolic syndrome has been connected to PM carriers, highlighting a potential role of the FMR1 gene in metabolism. Studies have shown that PM carriers tend to have higher waist circumference, glucose, and lipid levels, along with an increased prevalence of metabolic syndrome. Among carriers with FXTAS, fatigue is more pronounced compared to those without FXTAS and control subjects. Sleep apnea, frequently seen in FXTAS patients, can exacerbate chronic fatigue. Studies have documented chronic pain and fibromyalgia are more prevalent in women with the PM, both with and without FXTAS, compared to controls. Sleep disturbances are commonly reported among PM carriers even before neuropsychiatric symptoms emerge, with such issues being particularly noticeable among adult carrier daughters of men with FXTAS, who show a significantly higher incidence of sleep problems compared to controls. Finally, there is a broad spectrum of potential FXPACs in PM carriers, making it crucial to further explore and deepen our understanding of these conditions.

## P6 The impact of the focus on negative aspects of FXS: delivering a diagnosis in a positive, helpful way

### Jonathan Herring

#### Professor of Law, Faculty of Law, University of Oxford

*Orphanet Journal of Rare Diseases* 2025, **20(1)**: P6

This presentation will explore the ethical issues around delivery of a diagnosis of FXS. Most descriptions of FXS focus on a long list of “undesirable” consequences for the decision and a diagnosis of FXS is likely to focus on the negative impacts of the conditions. However, this paper will argue that such an approach is misconceived.

For many conditions it is understandable that the medical professional will focus on the negative aspects of the illness. Doing so will encourage the patient to take the proposed treatment so they can be returned to their “normal self” or as close to that as is possible.

But FXS is different from other conditions. First, there is no “cure” and “treatments” are limited. Focussing on the negative aspects to promote interventions is, therefore, unhelpful. Second, there are important strengths for those with FXS. Focussing on those and encouraging the person with FXS to use their special talents is more effective, rather than focussing on “negative” aspects. Third, for those with FXS, the FXS is a core part of who they are. It is tied up to their identity. To describe FXS solely in negative terms is harmful to their self-worth. Finally, those with FXS, although often presented in negative terms, have many strengths and much to teach those without the condition. To focus solely on the negative aspects of FXS is to lose out on learning those important lessons.

**Reference**

1. Herring, J., Johnson, K., Scerif, G., Weight, E., Richstein, J., Crawford, H., Robinson, H., Gawarammana, R., & Ellis, K. (2024). The joys of fragile X: Understanding the strengths of fragile X and delivering a diagnosis in a helpful, holistic way. Neurodiversity, 2. 10.1177/27546330241287685

## P7 Specificities and difficulty of X fragile genetic counselling in Arab societies: example of Morocco

### Siham C. Elalaoui, (MD, PhD)

#### ^1^Faculty of medicine and Pharmacy of Rabat, Mohamed V university, Rabat, Morocco. ^2^Medical Genetics Unit, Children’s Hospital, Rabat, Morocco

*Orphanet Journal of Rare Diseases* 2025, **20(1)**: P7

Fragile X syndrome is a genetic disorder, caused by the expansion of a CGG trinucleotide repeat on 5′ UTR of the fragile X messenger ribonucleoprotein-1 (*FMR1*) gene. Affected individuals have neurological, psychiatric, developmental problems, and some facial features. Fragile X syndrome (FXS) is an X-linked dominant disorder.

*FMR1* disorders range from premutation (PM) to full mutation (FM) leading to fragile X syndrome (FXS), fragile X-associated tremor/ataxia syndrome (FXTAS), and fragile X-associated primary ovarian insufficiency (FXPOI), according to number of repetitions. All males with FM have FXS, but only half of females with FM are clinically affected due to X-chromosome inactivation and mosaicism of *FMR1* variants (repeat size mosaicism and methylation mosaicism).

Arab countries have some specificities making the management and genetic counseling of FXS families so difficult and complex. Socio-economic status, education level of the parents, and accessibility to medical genetic consultation and genetic counseling services in Arab countries make this genetic counseling so difficult. Moreover, the fear of stigmatization and being labeled as having a child with genetic condition is hard to bear particularly for carrier females.

For these families especially those carrying a pre-mutation, the risk of developing FXTAS and FXPOI is difficult to explain and accept in many Arab societies. The recommendation to test all members of a family for the syndrome, even those without symptoms, is challenging in such societies due to misunderstanding and non-acceptance of the process and the implications of carrier screening.

## P8 Preconceptional fragile X screening

### Matthias Nybro Smith

#### Rigshospitalet, Copenhagen, Denmark

*Orphanet Journal of Rare Diseases* 2025, **20(1)**: P8

Fragile X syndrome, a prominent genetic cause of intellectual disability, presents distinct considerations for preconceptional screening. This talk will address the current status of preconceptional fragile X screening worldwide, examining where screening is offered and the guidelines that shape its use. Screening before conception enables prospective parents to make informed reproductive decisions and consider early interventions, offering advantages to those with family histories of fragile X or developmental disabilities. However, the approach also presents challenges, particularly the risk of identifying fragile X carriers with mild or asymptomatic forms of the disorder. Such unintended diagnoses, in individuals screened primarily for assessing reproductive risk, raise ethical concerns related to privacy, disclosure, and potential psychological impacts. These factors highlight the complexities of interpreting results within broader clinical and societal contexts. Cost-effectiveness, as well as logistical and ethical feasibility, remain points of debate for implementing routine screening on a larger scale. Additionally, questions arise regarding who should be screened, how results are best communicated, and whether specific public health strategies could address concerns of overdiagnosis while maximizing benefits. Ultimately, this discussion aims to underscore the need for thoughtful, evidence-based guidelines and international collaboration to ensure preconceptional fragile X screening can be integrated ethically and effectively, offering meaningful support to those at risk while respecting individual autonomy and choice.

## P9 Newborn screening for fragile X: past, present and future

### Don Bailey

#### RTI International, U.S.

*Orphanet Journal of Rare Diseases* 2025, **20(1)**: P9

Parents, clinicians, and researchers have long lamented persistent delays in diagnosing children with fragile X syndrome (FXS). Earlier identification could (a) reduce the lengthy, frustrating, and expensive process that families and children often endure when trying to understand the source of their child’s developmental challenges; (2) provide timely access to beneficial early intervention services; (3) help parents and clinicians tailor adapt more appropriately to their child’s special needs; and (4) enable access to support and advocacy groups.

Efforts to improve clinical detection (better recognition of signs and symptoms) are important but are unlikely to change the age of diagnosis, given that the symptoms of FXS overlap with many other developmental disorders. Genetic testing of children with any possible signs of FXS will accelerate accurate detection, but this means that diagnosed children would already be exhibiting developmental challenges.

Newborn screening (NBS)—testing babies shortly after birth for serious health problems—offers a public health platform within which screening for FXS could occur before symptoms appear. Although simple in concept, NBS for FXS continues to face multiple challenges, including (a) no proven treatment that must be provided early in life before symptoms appear; (b) the high cost of screening, counseling, and follow-up; and (c) social and ethical issues such as parental choice or the identification of FX premutation carriers.

This presentation addresses the history, status, and future possibilities of NBS for FXS. Our team has conducted two pilot studies and published numerous papers on various aspects of screening. Data from our most recently completed study will be presented. I will conclude with some thoughts about the future of early identification of children with FXS and how we might achieve that goal.

## P10 Newborn screening for fragile X (Australia)

### David E Godler^1,2,3^, Mohammed Alshawsh^1,4^, Jozef Gecz^5^, Ling Ling^1^, Mark Corbett^5^, Richard Saffery^1,2^, Dinusha Gamage^1^, Emma K Baker^1,2^, Minh Bui^6^, Michael J Field^7^, Carolyn Rogers^7^, James Pitt^8^,, Katrina Williams^4^, Sheena Arora^9^, Ronda Greaves^8^, David Francis^8^, Ralph Oertel^8^, Cas Simons^1^, Simon Sadedin^1^, Meg Wall^8^, Sebastian Lunke^8^, David J Amor^1,2^ Melissa Wake^1,2^

#### ^1^Murdoch Children’s Research Institute, Royal Children’s Hospital, Melbourne, Parkville, 3052, Australia. ^2^Faculty of Medicine, Dentistry and Health Sciences, Department of Paediatrics, University of Melbourne, Parkville, 3052, Australia. ^3^ E.D.G. Innovations and Consulting, St Kilda, 3004, Australia. ^4^Department of Paediatrics, Monash University, Melbourne, VIC, 3168, Australia. ^5^Robinson Research Institute and Adelaide Medical School, The University of Adelaide, Adelaide, South Australia, Australia. ^6^Centre for Epidemiology and Biostatistics, Melbourne School of Population and Global Health, University of Melbourne, Carlton, 3052, Australia. ^7^Genetics of Learning Disability Service, Hunter Genetics, Waratah, 2298, Australia. ^8^Victorian Clinical Genetics Services, Murdoch Children’s Research Institute, Royal Children’s Hospital, 1058, Melbourne, Australia. ^9^Centre for Health Economics Research and Evaluation, University of Technology Sydney, Broadway, NSW, Australia

##### Correspondence: David E Godler

*Orphanet Journal of Rare Diseases* 2025, **20(1)**: P10

Newborn screening using genomics offers the chance for children with rare diseases to receive new therapies from birth. One limitation is availability of genomic workflows in line with requirements for current newborn screening programs. We have developed a novel workflow called Epi-Genomic Newborn screening (EpiGNs) to meet these requirements. The EpiGNs uses one 3 mm newborn blood spot (NBS) punch for 1st-tier DNA methylation screen associated with selected imprinting and X-chromosome disorders. The shortlisted NBS positive by 1st tier screen are then analyzed using genomic and epigenetic testing on another 3 mm punch to confirm etiology. In 2022, EpiGNs program was funded by the Australian government to screen for fragile X, Prader Willi, Angelman, Dup15q, Turner, XXY, XXXY and XXYY syndromes, on NBS from 50,000 infants recruited as part of GenV—the whole-of-state birth cohort, and 50,000 infants consented for de-identified research in Victoria, Australia [1]. The key aims of the program are to scale the workflow and to determine prevalence from testing for these conditions at birth against GenV phenotypic measures collected up to 3 years of age. This presentation will describe program design, expected outcomes and their alignment with the criteria used in Australia for new conditions to be considered for standard of care newborn screening. Results will be presented from the initial pilot studies on newborn blood spots of 16,579 infants, focusing on 1st tier newborn screening for fragile X syndrome and confirmatory 2nd tier testing using *FMR1* CGG sizing and methylation analyses.

**Keywords:** *FMR1*, methylation, CGG expansion, newborn screening, full mutation.

1. https://www.mcri.edu.au/research/strategic-collaborations/centres/epi-genomic-newborn-screening-program 

## P11 Genetic testing for fragile X: challenges, experiences and results from University Clinical Centre Hospital in Gdańsk

### Karolina Śledzińska^1^, Kornelia Polat^2^, Anna Kłosowska^1^, Monika Cichoń-Kotek^1^, Ewa Kaczorowska^3^, Aleksandra Gintowt-Chakour^3^, Agata Żemajtys^1^, Beata Lipska-Ziętkiewicz^4^, Jolanta Wierzba^5^

#### ^1^Department of Pediatric, Hematology and Oncology, Medical University of Gdansk, Poland. ^2^5th year Medical Student, Medical University of Gdansk. ^3^Outpatient Genetic Clinic, University Clinical Centre, Gdansk, Poland. ^4^Clinical Genetics Unit, Department of Biology and Medical Genetics, Medical University of Gdansk, Poland. ^5^Division of Internal and Pediatric Nursing, Medical University of Gdansk, Poland

##### Correspondence: Karolina Śledzińska (ksledzinska@gumed.edu.pl)

*Orphanet Journal of Rare Diseases* 2025, **20(1)**: P11

**Introduction:** Fragile X syndrome (FXS) is considered one of the most common causes of intellectual disability (ID/DD) in males. We have analyzed the incidence of FXS among patients who visited Genetic Clinic. Furthermore, we performed a questionnaire of FXS patients’ carers around their satisfaction with medical care and quality of life, using the KIDSCREEN-10 instrument.

**Methods:** During the last 8 years, almost 40,000 patients, both children and adults, visited Genetic Clinic (GC) in the University Clinical Centre Hospital, Gdańsk, Poland. About 4000 patients were admitted due to ID/DD/autistic spectrum disorder based on ICD-10 diagnosis—ca 10% of total number. Annually, the number of patients admitted to GC increased, the same as the number of ID/DD/ASD patients, of whom the number increased from 6 to 12% of total number of patients. We analyzed the number of performed tests in case of suspicion of FXS.

**Results:** There were 1028 genetic tests for FXS performed (from 0 in 2015 to 238 in 2023), with 12 positive results. Additionally, 3 results were inconclusive, of whom patients were finally diagnosed with Klinefelter syndrome, and there were 2 girls—with full mutations (siblings of affected boys) and 3 with premutation. Medical information about 12 FXS cases will be presented. The results of the questionnaire revealed the good carers’ overall knowledge of the disease symptoms, genetic background and counselling, but underlined the need of more data regarding adulthood and maturity issues, together with transition period, lack of adequate multidisciplinary care, including psychological support.

**Conclusions:** The results show the complexity of ID diagnostic procedures, including FXS genetic testing. Furthermore, patients’ carers show dissatisfaction with current care, indicating the necessity of creating specialized centers of care. Therefore, after meeting with patients’ representatives we have organized the plan of providing satisfactory care in our hospital.

## P12 Ageing trajectories and dementia biomarkers in adults with FXS

### Hadassa, Kwetsie^1,2^; Sabine, Mous^3^; André, Rietman^3^; Agnies, Van Eeghen^1,2^

#### ^1^Emma’s Children’s Hospital, University of Amsterdam, Amsterdam, The Netherlands. ^2^Advisium,’s Heeren Loo Zorggroep, Amersfoort, The Netherlands. ^3^Department of Child & Adolescent Psychiatry/ENCORE Expertise center, Erasmus University Medical Center, 3000 CB, Rotterdam, The Netherlands

##### Correspondence: Hadassa, Kwetsie (h.r.kwetsie@amsterdamumc.nl)

*Orphanet Journal of Rare Diseases* 2025, **20(1)**: P12

**Rationale** Information on cognitive and adaptive ageing in adults with fragile x syndrome (FXS) is scarce and conflicting. Performing and interpreting neuropsychological assessment (NPA) is complex, while medical investigations such as lumbar puncture are invasive. Now that blood-based biomarkers of ageing diseases are increasingly becoming a reality, our knowledge on ageing in people with FXS might grow exponentially.

**Methods** We have evaluated the cognitive and adaptive ageing trajectories of 50 adult men and women with FXS. Individuals with prior cognitive or adaptive assessment in adulthood were included, and the same psychodiagnostic instruments to assess IQ or developmental age were used where possible. Clinical and genetic data were obtained from heathcare records. Blood samples were drawn and analysed for serum biomarkers of dementia pathology and general neurodegeneration, including p-Tau, NfL and GFAP.

**Results** Results of changes in cognitive and adaptive functioning will be shared along with the cross-sectional results of serum biomarker analysis. Potential risk factors of decline and/or neurodegeneration will be explored, including age, sex, level of premorbid functioning, comorbid disorders, and medication.

**Discussion** Longitudinal evaluation of cognitive and adaptive functioning in FXS is necessary to inform and prepare patients and caregivers for the future. Use of blood-based dementia biomarkers may show to be a cost-effective and less invasive diagnostic for this vulnerable medical population.

## P13 Nonsense mediated mRNA decay pathway and fragile X

### Natalie B. Tan^1,2,3^, Lachlan Jolly^4,5^, Michael Silk^7^, Saba Montazaribarforoushi^8^ Valeriya Gyurkovska^6^, UPF1 Consortium, David Ascher^7^, Nava Segev^6^, John Christodoulou^1,2,3^, Jozef Gecz^4,8^, Susan M. White^1,2,3^

#### ^1^Murdoch Children’s Research Institute, Parkville, VIC, Australia; ^2^Victorian Clinical Genetics Services, Parkville, VIC, Australia; ^3^Department of Paediatrics, The University of Melbourne, Parkville, VIC, Australia; ^4^Robinson Research Institute, ^5^School of Biomedicine, The University of Adelaide, Adelaide, SA, Australia; ^6^Biochemistry and Molecular Genetics, University of Illinois Chicago, IL, US, ^7^Structural Biology and Bioinformatics, Bio21 Institute, The University of Melbourne, Parkville, VIC, Australia; ^8^Adelaide Medical School, The University of Adelaide, Adelaide, SA, Australia

##### Correspondence: Jozef Gecz

*Orphanet Journal of Rare Diseases* 2025, **20(1)**: P13

**Background:** Nonsense mediated mRNA (NMD) decay pathway is dysregulated in Fragile X syndrome (FXS)^1,2^. FXS patient cells show hyperactivation of NMD as determined by elevated phosphorylation of UPF1 (“NMD active” P-UPF1). We previously studied NMD factors UPF3B^3^ and UPF2^4^ in neurodevelopment.

**Methods:** We recruited an international cohort of 23 individuals with de novo heterozygous missense variants in *UPF1* via the Matchmaker Exchange. We studied 5 patients with FXS.

**Results:** UPF1 individuals have variable cognitive impairment, dysmorphism and malformations. Variants reside within C-terminal domains. 3D modelling predicts impact on ATP-dependent helicase activity. Western blots of patient (n = 6) and control (n = 6) LCLs showed increase in P-UPF1, decreased UPF3B and compensatory increase in UPF3A. Differentially expressed genes (DEGs) significantly overlapped and correlated (R = 0.95) with those of FXS LCLs. These DEGs did not correlate with DEGs of UPF3B and UPF2 LCLs.

**Discussion:** Our data suggest that variants located in the UPF1 ATPase interface lead to altered UPF1 function. UPF1/FXS patient DEGs are distinct from UPF3B/UPF2 LCL NMD DEGs. The data suggest mechanism of UPF1/FXS may not be just hyperactive NMD.

**Refs: 1.** Kurosaki T, et al. Nat Cell Biol. 23(1):40–48, 2021; **2.** Kurosaki T, et al. Genome Biol. 25(1):31, 2024; **3.** Tarpey P, et al. Nat Genet. 39(9):1127–33, 2007; **4.** Johnson JL, et al. Neuron. 104(4):665–679, 2019.

**UPF1 Consortium:** SA Ahmed, HA Saif, I Anselm, L Armstrong, J Baker, GB Ferrero, K Bell, M Bertrand, A Brusco, H-M Büttel, A Chapin, J Chenbhanich, M Chung, H Cox, F Demurger, C Dubourg, JI Estrada-Veras, T Haack, G Houge, S Jurgensmeyer, S Kaspar, R Keller, F Kortuem, G Lemire, G Lesca, M Morrow, C Netzer, A O'Donnell-Luria, M O'Leary, J Picker, V Pullano, K Rawson, L Rodan, M Rossi, A Roston, S Spranger, D Stevenson, Stuurman, A Tzschach, C Velmans, D Viskochil, R Woitschach.

## P14 Getting personal in a CBD-trial: individualized trial design and outcome measures

### Dr. Agnies M. van Eeghen, MD, PhD

#### Intellectual Disability Physician, Amsterdam University Medical Centers/’s Heeren Loo

*Orphanet Journal of Rare Diseases* 2025, **20(1)**: P14

**Rationale:** There is great interest for interventional studies in FXS, in order to improve care and wellbeing of individuals with FXS and their families. Thus far most trials showed negative results, which may be due to the proposed therapy, but possibly also due to limited validity of currently used outcome measures. Additionally, subgroups or single participants could benefit, who may not be identified on a group level. This may result in individuals with FXS missing out on potentially effective therapies.

To address these issues, there is increasing interest in designing trials that provide evidence on an individual level, as well as on a group level. Also, individualized outcome measures are underway which may be more valid and simultaneously provide more information on the clinical and social relevance of the proposed therapies.

In this presentation, the design of an upcoming cannabidiol-trial is presented to discuss innovative trial methodology such as N-of-1 trials and individualized outcome measures for individuals with FXS.

## P15 Medications for fragile X syndrome: current use and (near) future horizons

### Dr Andrew C. Stanfield

#### Patrick Wild Centre, University of Edinburgh, 23 Tipperlinn Road, Edinburgh, UK, EH10 5HF

*Orphanet Journal of Rare Diseases* 2025, **20(1)**: P15

Good management of individuals with fragile X syndrome (FXS) involves well co-ordinated multidisciplinary care. Medications can be useful for some people with FXS as part of this multidisciplinary approach, but it is important that they are prescribed appropriately. This review aims to summarise the current evidence for medications used commonly in FXS and highlight potential future developments.

The most common ‘supportive’ medications in use are selective serotonin reuptake inhibitors (SSRIs), stimulants and antipsychotics. The evidence for these medications is primarily derived from studies in non-fragile X populations. One placebo controlled trial has suggested that sertraline may improve some measures of cognition in young children, while two small studies have reported potential benefits of stimulants to children with FXS. Unblinded, observational studies in clinic populations suggest around 70–75% of individuals show benefit from these medications.

Increasing numbers of studies are investigating medications targeted at the underlying mechanism of fragile X. Some of these are repurposed medications (e.g. minocycline, metformin) while others are novel medications (e.g. arbaclofen, cannabidiol gel, zatolmilast). At present, the best evidence exists for minocycline on the basis of a single medium sized trial which showed both clinical improvement and biomarker engagement. Arbaclofen may also be helpful in younger children, based on a re-analysis of a previous large Phase III trial, but new prospective studies are required to confirm this. The results of definitive trials are currently awaited for metformin, cannabidiol and zatolmilast.

At present, there exists little fragile X specific evidence to make treatment recommendations. It is pragmatic to consider trials of SSRIs and stimulants when indicated, with antipsychotics for agitation potentially considered on a short-term basis where other interventions have failed. Targeted interventions require further study before any can be definitively recommended. All interventions should be part of a wider multidisciplinary care plan.

## P16 The role of allelic instability and mosaicism in *FMR1* premutation

### Flora Tassone

#### Department of Biochemistry and Molecular Medicine, University of California Davis, School of Medicine, Sacramento, 95817 CA, USA. MIND Institute, University of California Davis Medical Center, Sacramento, 95817 CA, USA

*Orphanet Journal of Rare Diseases* 2025, **20(1)**: P16

**Background:** Individual carriers of the *FMR1* premutation (PM, CGG between 55 and 200) have a significant health risk of developing the *FMR1* premutation associated conditions (FXPAC) over their life span. Although mosaicism is well described in individuals with the full mutation affected by fragile X syndrome, recent studies have demonstrated the presence of CGG allele somatic instability in individuals with a PM.

**Method:** CGG repeat number, AR, AGG interruptions, *FMR1* mRNA expression levels, and measures of CGG allele instability were measured by Southern blot and PCR analysis. Ten SNPs within DNA repair genes were investigated to determine their potential association with somatic instability.

**Results:** 90% and 70% of female (n = 426) and male (n = 421) participants showed *FMR1* allelic instability. We observed a positive correlation between CGG repeat size and *FMR1* mRNA expression levels, and a negative between AGG interruptions and allele instability. We also observed increased levels of somatic expansion CGG size and AGG dependent. Changes in the extent of blood somatic expansion were observed over time and were limited to PM alleles carried on the active X chromosome. 33% of the female participants and 45% of the male participants displayed a higher increase of the larger-CGG sized allele over time. Finally, we demonstrate a correlation between SNPs associated with the DNA repair genes, FAN1 and MSH3 and allele instability.

**Conclusion:** Longitudinal studies are needed to fully understand how somatic mosaicism may impact the development and brain function in PM over time. Although some phenotypic PM features are present in childhood, particularly in boys the majority of clinical problems in carriers have an onset in adulthood (FXPOI and FXAND) or in aging (FXTAS). A role of variants in DNA repair genes could affects disease onset and severity as observed in other repeat expansion diseases.

## P17 Early predictors and outcomes of strong attention in fragile X syndrome

### Gaia Scerif

#### Attention, Brain and Cognitive Development Group, Department of Experimental Psychology, University of Oxford, United Kingdom

*Orphanet Journal of Rare Diseases* 2025, **20(1)**: P17

**Background:** Attention problems can be an area of real difficulty for older children, young people and adults with Fragile X Syndrome (FXS), but these difficulties are highly variable, with some people experiencing extreme problems, and others encountering fewer challenges. Multiple factors may account for the emergence of these differences between older people with FXS, and may offer potential targets for early support. We focus on cognitive factors that predict this variability in attention, and in turn on how attention might influence differences in later outcomes.

**Objective:** To understand differences in attention in FXS, and its longitudinal outcomes.

**Method:** Using a prospective longitudinal approach, we investigated: (1) early attention profiles and communication for boys with FXS (N = 59), in comparison with children without genetic conditions; (2) characteristics associated with stronger attention skills; and (3) the extent to which early attentional differences predicted communication across these children 12 and 24 months later.

**Results:** At the group level, we know that children with FXS show difficulties in attention, communication, and numeracy. However, our data suggest a high degree of variability in attention between boys with FXS, and differences in attention between boys with FXS predicted their communication development longitudinally.

**Conclusions:** Early attentional differences are striking across boys with FXS, with multiple factors contributing to them. Attention differences contribute to differences between children with FXS in their emerging communicative skills, and we hypothesise a parallel relation to numeracy. The predictive role of attentional processes suggests that we could focus on intervening on both attention and other outcomes as they emerge. We close with evidence-based recommendations for how integrated attention interventions may be optimized.

**Acknowledgments:** Prof Scerif's contribution was supported by ESRC UKRI grant ES/X013561/1.

## P18 AI and gait analysis in the assessment of motor skills in FXS

### Dr. Zimi Sawacha

#### University of Padova, Italy

*Orphanet Journal of Rare Diseases* 2025, **20(1)**: P18

In Fragile X Syndrome (FXS) children the most common musculoskeletal manifestations are flexible flat feet, hypotonia and excessive joint laxity, which may cause the presence of non-physiological gait patterns. Previous gait analysis studies have evidenced significant alterations both in terms of lower limb joint angles and muscle activity. Within this context, the possibility to identify specific characteristics that can be associated with FXS from gait analysis data has recently attracted attention.

In the present contribution, preliminary results of a collaboration (2018–2024) between the BiomovLab and the Fragile X Center of the University of Padova are reported. The project aims at investigating the feasibility of stratifying FXS children not only based on their classical clinical and molecular profiles but also based on their functional capabilities. A dataset of 80 subjects has been collected over the years, including data from typically developing and FXS children (i.e. full mutation with and without mosaicism and premutation). Multiple gait trials have been recorded at self-selected speed using 4 synchronized cameras and a surface electromyographic system; joint kinematics and muscle activity of 4 lower limb muscles have been analyzed. For each child a total of 124 features have been made available. Different machine learning algorithms (i.e. supervised and unsupervised) have been applied in combination or not with Dynamic Time Warping analysis. Preliminary results show that muscle activity features perform better in terms of accuracy, while joint angles yield greater consistency. Further investigations will explore alternative approaches with supervised model structures.

## P19 Cognitive and environmental factors contributing to social strengths and challenges in people with fragile X syndrome

### Katherine Ellis

#### University College London, UK

*Orphanet Journal of Rare Diseases* 2025, **20(1)**: P19

Sociability in people with fragile X syndrome (FXS) is characterised by a juxtaposition of challenges (e.g. social anxiety) and strengths (e.g. social motivation). Our work describes nuanced profiles of strengths and challenges in social cognitive skills in those with FXS, which may underpin their unique behavioural phenotype. During a passive viewing gaze following paradigm, whilst children with FXS spent significantly less time looking at the cued object, they shifted their initial gaze towards the object, following the agent’s cue, as frequently as neurotypical children. In addition, whilst as many children with FXS overimitate (a key skill for social learning and social affiliation) as neurotypical children, they do so less frequently. Findings do not indicate a difficulty in social cognition per se, but perhaps that challenges in attention or working memory impede children from fully engaging and learning from their social environment. Supporting this, many children with FXS show good performance on implicit mentalizing tasks, which remove attention and working memory demands associated with poor explicit mentalizing task performance in this group. Understanding these neurocognitive profiles may inform interventions that harness individual’s current abilities (e.g. social motivation, mentalizing) and supportive social environments. We have found that parents of autistic children who use a variety of scaffolding techniques support autistic children to re-engage as quickly as neurotypical children following disengagement from social interaction. Children with FXS may also benefit from supportive social partners, and other adaptations to the social environment.

## P20 Cognitive-behavioral therapy with adolescents and young adults with FXS

### Federica Alice Maria Montanaro^1,2^

#### ^1^Child and Adolescent Neuropsychiatry Unit, Bambino Gesù Children's Hospital, IRCCS, Rome, 00165, Italy. ^2^Department of Education, Psychology, Communication, University of Bari Aldo Moro, Bari, 70122, Italy

*Orphanet Journal of Rare Diseases* 2025, **20(1)**: P20

**Background:** Fragile X Syndrome (FXS) is an X-linked neurodevelopmental disorder leading to intellectual disability (ID) along with cognitive-behavioral difficulties. Despite clear understanding of the cognitive-behavioral phenotype of FXS and the need for tailored interventions, empirical research on the effectiveness of behavioral treatments for patients with FXS remains limited, especially studies focused on adolescents and young adults.

**Method:** This work provides empirical evidence of a combined neuropsychological and cognitive behavioral group therapy (nCBT) for the treatment of adolescents and young adults with FXS. Specifically, it presents "Corposamente" (CoM)^1,2^, a group nCBT program conducted with ten young adults with FXS, for which standardized measures were collected before and after the intervention. Additionally, this study presents pilot data from an ongoing randomized controlled trial involving adolescents with FXS, which includes structured evaluations conducted pre- and post-treatment.

**Results:** The results indicate that nCBT can effectively reduce anxiety symptoms, enhance executive functioning, and facilitate the acquisition of new socio-relational skills, ultimately leading to improvements in adaptive functioning and quality of life among adolescents and young adults with FXS.

**Conclusions:** Given that CBT is already established as a first-line treatment for anxiety and depression disorders, and with emerging evidence supporting its effectiveness for emotional disorders in individuals with ID, the integration of cognitive and neuropsychological interventions for treating clinical manifestations associated with FXS appears to be a promising approach worth further pursuit.

**References**

1. Montanaro, F. A. M., Alfieri, P., & Vicari, S. (2023). "Corp-Osa-Mente", a Combined Psychosocial-Neuropsychological Intervention for Adolescents and Young Adults with Fragile X Syndrome: An Explorative Study. Brain sciences, 13(2), 277.
2. Montanaro, F.A.M., Alfieri, P., Caciolo, C., Spano, G., Bosco, A., Vicari, S. (2024). Effects of a combined neuropsychological and cognitive behavioral group therapy on young adults with Fragile X Syndrome: an explorative study.Research in developmental disabilities, 154, 104839. 10.1016/j.ridd.2024.104839.

## P21 Social inhibition across early childhood in fragile X syndrome

### Abigail L. Hogan, PhD, & Jane E. Roberts, PhD

*Orphanet Journal of Rare Diseases* 2025, **20(1)**: P21

**Background:** Social inhibition is a temperament profile characterized by fear and withdrawal in social situations. In neurotypical (NT) children, elevated social inhibition during preschool predicts social anxiety symptoms and diagnoses later in development. Social anxiety affects many individuals with fragile X syndrome (FXS) and significantly impacts daily functioning and quality of life. However, little is understood about the early predictors of social anxiety in FXS.

**Objectives:** To characterize developmental change in social inhibition across early childhood and determine whether early social inhibition predicts later social anxiety symptoms in FXS.

**Method:** Participants included 54 children with FXS (38 males) and 38 NT children (31 males) assessed longitudinally from 3 to 7 years of age, for a total of 232 observations. Social inhibition was measured via (a) a composite score of behaviors observed during a Stranger Approach task, and (b) the Shyness subscale score from the Children’s Behavior Questionnaire (CBQ). At the final assessment, social anxiety symptoms were assessed via the Preschool Anxiety Scale-Revised (PAS-R).

**Results:** For the Stranger Approach, the age*group interaction was significant, *F*(1, 79.5) = 4.28, *p* = 0.042, in that social inhibition decreased across age in the NT group while remaining steady in FXS, *B* = 0.69. For CBQ Shyness subscale, there were main effects of age, *F*(1, 56.4) = 5.04, *p* = 0.029, *B* = -0.13 and group, *F*(1, 143) = 4.84, *p* = 0.029, *B* = 0.81, with higher social inhibition across early childhood in FXS. Within FXS, CBQ Shyness intercept (at 3.81 years) and slope predicted later social anxiety symptoms, *F*s ≥ 4.63, *p*s ≤ 0.037.

**Conclusions:** Results suggest that social inhibition, a known risk factor for social anxiety, is elevated across early childhood and associated with later social anxiety symptoms in FXS. These findings are critical to better understanding the early predictors of social anxiety in FXS, which will in turn facilitate early identification and inform preventative treatment efforts.

## P22 Sleep problems in Fragile X syndrome (FXS)

### Dück A., Reis O., van Treeck L., Kölch M.

#### Rostock University Medical Center, Department of Child and Adolescent Psychiatry and Neurology

*Orphanet Journal of Rare Diseases* 2025, **20(1)**: P22

**Abstract** It is known that up to 77% of children and adolescents with FXS suffer from sleep disorders (Richdale et al. 2003, Kidd 2014), which are often a considerable burden for families. Up to 64% of the FXS affected children receive medication (Kronk et al. 2010). Disturbances in sleep architecture and circadian rhythmicity are known and appear to be strongly age-dependent. Comparable data from objective measurement methods are not available despite the considerable need.

At the same time, up to 50% of FXS affected persons show neurodiverse phenomena from the autism spectrum. During puberty, symptom complexes such as ADHD and/or depressive symptoms often occur. These pose additional major challenges for the children and adolescents and their families (Wheeler et al. 2013).

The lecture will present and discuss the latest findings on the connection between sleep, development and mental disorders. To investigate this relationship, our research group has adapted a minimally invasive yet complex setting for investigating sleep and circadian rhythmicity to the individual needs of FXS affected persons and their families. It includes saliva samples for the determination of melatonin and cortisol, actigraphy for the measurement of movement and light, a sleep diary, and polysomnography. This completely mobile setting, which can be used anywhere, will also be presented in detail in the lecture. The feasibility of the setting was examined using two groups of FXS affected persons in Germany (Rostock, Mecklenburg-Western Pomerania and Amberg, Bavaria) and was very well tolerated by the individuals and their families or care systems.

## P23 Routinely-acquired data in the United Kingdom: studies in fragile X syndrome

### Presenting Author: Andrew McKechanie; McKechanie A.G.^1,2,3^, Stanfield A.C.^1,2,3^, Fisher L.^4^, Morgan C.L.I.^4^, Jones B.I.^4^, Cooper A.^5^, Conway P.^5^

#### ^1^The Patrick Wild Centre, University of Edinburgh, Edinburgh, UK. ^2^Simons Initiative for the Developing Brain, University of Edinburgh, Edinburgh, UK. ^3^NHS Lothian, Edinburgh, UK. ^4^Human Data Sciences, Cardiff, UK. ^5^Shionogi, London, UK

*Orphanet Journal of Rare Diseases* 2025, **20(1)**: P23

**Background:** In this series of studies, we used large-scale, routinely-acquired healthcare data from England to examine questions relating to pre-diagnostic healthcare, epidemiology, healthcare utilisation and mortality in fragile X syndrome (FXS) in larger samples than is often possible when relying on cohorts acquired by individual clinicians or centres.

**Methods:** We used linked data from the Clinical Practice Research Datalink Aurum database which contains longitudinal (30+ year) data on ~ 38 million patients and linked data from the Hospital Episodes Statistics and Office for National Statistics (ONS) mortality datasets. Cases were selected if they had ≥ 1 medical code indicative of FXS. Patients with FXS were matched on a 1:1 ratio to non-FXS controls on age, gender and practice.

**Results:** The highest observed prevalence of FXS across age groups was ~ 1/2400 for males ~ 1/6800 for females. We estimated that the prevalent population of individuals with a FXS diagnosis in the UK is 6835, with a further 9828 not yet being diagnosed. Prior to diagnosis, individuals with FXS had significantly higher numbers of healthcare contacts in all settings (primary care, in-patient, out-patient and Accident & Emergency) than controls. Compared to matched controls, individuals with FXS had a significantly higher risk of death (Hazard Ratio 2.03) and a mean of 10 life-years lost.

**Conclusion:** The use of large-scale, routinely-acquired data can provide insights into the health and healthcare of individuals with fragile X syndrome. This approach should be considered in other genetic conditions.

## P24 Multidisciplinary care at the FXS Centre Barcelona

### Dr. Ana Roche

#### Division of Pediatric Neurology, Parc Taulí Hospital Universitari. Institut d’Investigació i Innovació Parc Taulí (I3PT-CERCA)

*Orphanet Journal of Rare Diseases* 2025, **20(1)**: P24

The *Corporació Sanitària Parc Taulí (CSPT)* is a set of different centers that offer the whole spectrum of services to cover healthcare needs of the individuals and families of its referral area. It is located in Sabadell, 12 miles north-west of Barcelona, and provides healthcare services from newborn to adulthood.

Our unit for cognitive and behavioral disorders of genetic origin became years ago a reference center for rare disorders such as Angelman Syndrome, Prader Willi and Fragile X in Spain.

Multidisciplinary care for FXS is crucial for families, who have the opportunity to receive medical (neurology, genetics, orthopedy, cardiology, psychiatry) and psychological advice from experienced professionals in the same day. This monographic day becomes also a “meeting point” where some families share their experiences. Also, individuals with premutation (mothers, grandfathers, etc.) are followed at our Neurology Clinic.

Constant communication among the different professionals and a fluid dialog of the reference health caregivers (pediatric neurologist and psychologist) with families has been a key to growing our multidisciplinary FXS Unit and get us ready to participate in research and clinical trials.

## P25 The management of children with X fragile syndrome: experience from a neuropsychiatric center in Italy

### Dr. Paolo Alfieri

#### Child and Adolescent Neuropsychiatry Unit, Department of Neuroscience, Bambino Gesù Children’s Hospital, IRCCS, Rome, Italy

*Orphanet Journal of Rare Diseases* 2025, **20(1)**: P25

Fragile X syndrome (FXS) is the most common genetic condition leading to intellectual disability and is associated with a distinct cognitive-behavioral phenotype, as well as an increased risk for neurological and medical problems.

This work summarizes the experience in the management of children with FXS at Bambino Gesù Children’s Hospital, a specialized pediatric center in Italy. Specifically, it presents a review of a 5 years’ work in which 53 children with FXS were monitored and treated using a multidisciplinary approach involving child neuropsychiatrists, psychologists and speech therapists. Key elements of management included accurate diagnosis, personalized medical and therapeutic interventions, and robust family support systems.

Furthermore, with the aim to build a family-centered clinic, our team conducted a national survey^1^ on main symptomatology and treatment priorities of children with FXS. The survey was compiled by one hundred and thirty-eight people, including family members, clinicians and individuals with FXS themselves. The results enabled a deeper understanding of the primary challenges and treatment needs from the families' perspectives, thereby facilitating the development of more tailored interventions. Specifically, in response to continued feedback from families, we created a specialized center in which different interventions were implemented. Therapeutic strategies encompassed pharmacological treatments for associated conditions such as ADHD and anxiety, comprehensive cognitive-behavioral therapy for adolescents with FXS, tailored families psychoeducation, and speech and language psychoeducation.

Collected data revealed the efficacy of the integrated care model, emphasizing collaboration across various disciplines to address the multifaceted needs of children with FXS.

**Reference**

1. Montanaro, F. A. M., Alfieri, P., Caciolo, C., Brunetti, A., Airoldi, A., de Florio, A., Tinella, L., Bosco, A., & Vicari, S. (2024). Fragile X Syndrome and FMR1 premutation: results from a survey on associated conditions and treatment priorities in Italy. *Orphanet journal of rare diseases*, *19*(1), 264. https://doi.org/10.1186/s13023-024-03272-0

## P26 Fragile X-care at paradise city…. Maulbronn Children’s Hospital

### Dr. Ulrike Gaiser

#### Kinderzentrum Maulbronn, Maulbronn, Germany

*Orphanet Journal of Rare Diseases* 2025, **20(1)**: P26

*A unique way of collaboration of therapists to support fragile X families*

Welcome to a little paradise in caring about Fragile X families with children in the early years! This is where therapists of all disciplines, psychologists, social workers and medical doctors all work together in an in-patient setting in southern Germany in what we call “intensive social paediatric care”.

We do care about daily life problems such as behavioral difficulties, addictive media consumption, eating disorders and other impacts on family life. Our inpatient program takes place whenever outpatient facilities are not sufficient—be it that there is a professional 24/7—setting needed or be it the need of increased therapeutical intensity. The basics of our work are behavioural analysis, parent–child interaction, systemic psychotherapy and an interdisciplinary team setting will be presented.

We will talk about our work with focus on eating problems, behavioural problems and increased media consumption.

The need of exact diagnosis of the developmental level will be discussed as well as possibilities to close gaps in development such as alternatives to spoken language; visual ancillaries and useful daily helpers.

## P27 Presentation and functional overview of the fragile X Center in Padua Italy

### Zimi Sawacha^1^, Alessandra Murgia^2,3^

#### ^1^Department of Information Engineering, University of Padova, Italy. ^2^Department of Women’s and Children’s Health, University of Padova School of Medicine, Padova, Italy. ^3^Pediatric Research Institute Città della Speranza, Padova, Italy.

*Orphanet Journal of Rare Diseases* 2025, **20(1)**: P27

Fragile X conditions define a family of seemingly unrelated clinical disorders that despite a monogenic etiology, due to opposite pathogenic mechanisms, display a wide array of different clinical manifestations.

Many are the clinical needs of children and families who live with Fragile X Syndrome and related conditions, and these needs pose several clinical questions requiring informed and timely professional answers. Due to the complex nature of these disorders, individual answers, even of high professional quality, may not be enough, and a multidisciplinary integrated strategy seems much more appropriate.

We will present the activity of the Fragile X center of the University Hospital of Padua. This is established on a pediatric core, historically and functionally founded upon state-of the art FMR1 molecular diagnostics but fed and grown due to the strong multidisciplinary clinical program developed in the course of about ten years. The combination of these two integral and inseparable components, which has and continues to generate research activity, has allowed the Center to become a recognized national molecular and clinical diagnostic referral center. The Padua Fragile X Center, which is largely dedicated to subjects in the developmental age, integrates all the subspecialties involved in the care of the neurodevelopmental problems, as well as of the pediatric comorbidities typical of Fragile X syndrome. The whole clinical activity of the Center is supported by a strong organizational infrastructure that, while assuring the function of a dedicated team within the complex routine of a children’s hospital, keeps the children and their families, coming from all over the Italian territory (40% from the local territory; 60% from other Italian regions), at the center of attention and care. This large professional team is also actively involved in national and international research, particularly focused on the study of very early mechanisms leading to loss of function or cell toxicity in Fragile X disorders, and on the characteristics of motor control and muscle activity in children with Fragile X syndrome.

## P28 Abstract for the workshop: navigating diagnoses in fragile X syndrome

### André Rietman^1^, Bram Dierckx^2^

#### ^1^Health Care Psychologist, Erasmus MC. ^2^Child and adolescent Psychiatrist, Erasmus MC

*Orphanet Journal of Rare Diseases* 2025, **20(1)**: P28

When individuals are diagnosed with Fragile X Syndrome (FXS), the diagnosis alone doesn't capture the full picture. Psychological and psychiatric assessments are vital for identifying specific needs for treatment and support, but they often introduce additional labels.

As psychiatrists and psychologists, we frequently struggle with how to communicate these diagnoses to parents. Should we use terms like “severe intellectual disability” with parents of young children? If a child already has an autism diagnosis, is it necessary to add labels like ADHD or ODD? Or should we strictly follow the DSM-5 manual to determine if a disorder is present?

Another key consideration is addressing the feelings of loss and grief that parents may experience following a diagnosis. While labels can facilitate access to necessary services and support, they can also be overwhelming and distressing for families.

At our national FXS center of expertise in the Netherlands, we provide comprehensive care for children with FXS and their families. Our research focuses on the cognitive and mental functioning of children with FXS, including long-term follow-up studies. Our goal is to balance the need for precise diagnosis with sensitivity to the emotional impact on families, ensuring an approach that is both clinically effective and compassionate. Through open communication and support, we strive to help families navigate the complexities of FXS with the best possible care and understanding.

Join this interactive workshop to discuss strategies for conveying FXS diagnoses to parents and supporting families through their journey.

## P29 Gene therapy in fragile X syndrome: a caregiver perspective

### Sarah EA Eley, Sydni Weissgold, Andrew C Stanfield

#### Patrick Wild Centre, University of Edinburgh, Edinburgh, UK

*Orphanet Journal of Rare Diseases* 2025, **20(1)**: P29

**Background:** There have been increasing numbers of clinical trials of medications for fragile X syndrome (FXS) in recent years. As yet none of these have led to widespread changes in clinical practice. Genetic therapies represent a different therapeutic approach, which aim to address the genetic mechanisms by which FXS arises. This is an area of increasing research importance in neurodevelopmental conditions. We are aware that for some people this could be a controversial subject, so we think it is important that families affected by FXS get the chance to give their views about future genetic therapies.

**Objective:** Collect caregivers’ views on the use of gene therapy in FXS.

**Methods:** We developed a questionnaire, alongside a group of parents/caregivers of a child with FXS to ensure the language used was appropriate and that it would allow a variety of views to be captured. It contained questions around current knowledge, what families think of gene therapy and their views on gene therapy trials taking place. Responses were analysed by thematic analysis.

**Results:** There were 195 responses. 21% of respondents’ dependants had previously been involved in a clinical trial. 60.5% of responses were from the UK, 8.2% from the rest of Europe, 22.1% were from the Americas, 3.1% from Australia/NZ and 2.1% from rest of world.

Data from the questionnaires were grouped into themes and subthemes. The three themes that emerged from the data were quality of life, outcomes and feelings.

**Conclusion:** The findings showed a strong interest from the Fragile X community in gene therapy trials taking place and the impact it could have on their family. There was some trepidation about unintended consequences, the newness of the treatment and tolerability. Overall, though, caregivers felt hopeful, excited and interested in the prospect of gene therapy potentially providing a new treatment option.

## P30 Somatic mosaicism of unmethylated FMR1 full mutation and heterogeneous premutation alleles in an asymptomatic older male

### R. Polli^1,2^, E. Bettella^1,2^, M, Cameran^1,2^, V. Liani^1^, E. Cavaliere^1^, F. Spolaor^1^, A. Guiotto^3^, G. Bonato^4^, M. Carecchio^4^, G. Mioni^5^, E. Di Giorgio^6^, Z. Sawacha^3^, A. Murgia^1,2^

#### ^1^Department of Women’s and Children’s Health, University of Padova, Italy. ^2^Pediatric Research Institute “Città della Speranza”, Padova, Italy. ^3^Department of Information Engineering, University of Padova, Italy. ^4^Department of Neuroscience, University of Padova, Italy. ^5^Department of General Psychology, University of Padova, Italy. ^6^Department of Developmental Psychology and Socialization, University of Padova, Italy

*Orphanet Journal of Rare Diseases* 2025, **20(1)**: P30

**Background**: Fragile X syndrome (FXS) is the most common inherited cause of intellectual disability and autism, caused by full mutations (FM) of the FMR1 gene with expansions of over 200 CGG repeats in 5'UTR region. These expansions lead to promoter methylation, resulting in transcriptional silencing and loss of the FMRP protein. Premutations (PM), with 55–200 CGG repeats, remain transcriptionally active but cause toxic overexpression, leading to premutationassociated conditions (FXPAC). Rare cases of FXS with little or no methylation show residual gene function, associated with better cognitive outcomes. Complex patterns of methylation and mosaicism increase the risk of FXPAC.

**Objective:** A thorough clinical evaluation was performed on a 77-year-old asymptomatic male, subjected to FX genetic testing in the context of a segregation analysis, who was found to have a complex and rare molecular mosaicism, with both FM and a wide range of premutation alleles, all unmethylated.

**Methods:** A comprehensive clinical assessment was conducted, including neurological exams and standardized neuropsychological tests (WAIS-IV, MMSE) to assess IQ, cognitive deficits, and functions such as language, memory, attention, visuospatial abilities, and executive functions. Gait analysis and surface electromyography (sEMG) were also performed. A brain MRI was performed to check for possible degenerative signs, and RNAseq was carried out to thoroughly characterize FMR1 gene function.

**Results:** Neuropsychological evaluation revealed normal or higher-than-normal performance in several cognitive domains, with balance disturbances and abnormalities in posture, ground reaction forces, muscle activity, and joint angles. No other neurological signs were observed. Preliminary RNAseq data showed increased FMR1 mRNA levels and identified differentially expressed genes involved in neuronal function, synaptic plasticity, and cellular stress response.

**Conclusion:** Investigating these rare cases could reveal genetic and functional mechanisms that protect against clinical manifestations, providing important insights into factors that modulate the wide phenotypic variability in both FXS and FXPAC.

**Grants:** *This work was supported by: FRAXA Research Foundation Fellowship, PRIN 20227JA8R3.*

*BIRD-SID 225494-2022, IRP (Pediatrics Research Istitute “Città della speranza”) RESEARCH GRANTS 2024-2026 IRD-SID*

*225494-2022, IRP (Pediatrics Research Istitute “Città della speranza”) RESEARCH GRANTS 2024-2026*

## P31 Importance of updating old genetic diagnoses to offer proper personalized follow up, treatment and genetic counselling in *FMR1* related conditions

### Neus Baena^1^, Carmen Manso^1^, Marta Rubio^2^, Lorena Joga^3^, Ariadna Ramírez^3^ and Ana Roche^3^

#### ^1^Division of Genomic Medicine, Parc Taulí Hospital Universitari. Institut d’Investigació i Innovació Parc Taulí (I3PT-CERCA). ^2^Division of Neurology, Parc Taulí Hospital Universitari. Institut d’Investigació i Innovació Parc Taulí (I3PT-CERCA). ^3^Division of Pediatric Neurology, Parc Taulí Hospital Universitari. Institut d’Investigació i Innovació Parc Taulí (I3PT-CERCA)

*Orphanet Journal of Rare Diseases* 2025, **20(1)**: P31

Fragile X syndrome (FXS) is the main cause of intellectual disability and autism of inherited genetic origin, due to the expansion of the promotor region of *Fragile X Messenger Ribonucleoprotein 1  (FMR1)*. In recent years, the accuracy of genetic testing has greatly improved, including our ability to detect the number of CGG triplet repeats in the diagnosis of *FMR1* related disorders. This increased diagnostic accuracy is helping to outline new diagnoses and improve counseling and treatment in families with a history of FXS, especially when clinical signs differ from the expected.

We present a group of families who were followed up in our rare disease unit due to a diagnosis of FXS in at least one family member, performed during the 1990s either in our center or in a different one, mainly with Southern Blot Technique.

Some of the affected members of these families, although classified as FXS, had a different evolution than expected compared to other families, such as milder symptoms in full mutation (FM) male carriers, or absence of anxiety in FM female carriers, so the genetic study was repeated by our lab with updated techniques (RT-PCR).

This way, we have proved that several patients with FXS full mutation (FM) diagnosis were actually mosaics for FM and premutation (PM), others were PM carriers instead of FM carriers, and a 3rd group were FM carriers (a woman with childbearing desire, an unmethylated FM man) instead of PM carriers.

Reviewing and updating old genetic diagnoses guided by clinical unexpected (milder) evolution and offering genetic counseling to families improves personalized follow-ups, customized treatments and setting realistic expectations for offspring.

## P32 Discerning links between speech and language with executive functioning and tremor in individuals with the *FMR1* premutation

### Nell Maltman, PhD

#### University of Arizona Department of Speech, Language, and Hearing Sciences

*Orphanet Journal of Rare Diseases* 2025, **20(1)**: P32

**Background/objective**: Speech and language rely on executive functioning and motor processes. Emerging evidence suggests speech and language production may be associated with the FXTAS clinical phenotype, though how these are linked prior to FXTAS onset is unclear. This study had two aims: (1) Assess group differences in standard measures of speech and language; (2) Examine links between speech and language with executive functioning and tremor in individuals with and without the *FMR1* premutation.

**Methods**: Participants included 42 individuals with the *FMR1* premutation (31F, 11M) and 41 controls (24F, 17M), who completed a 2-h virtual visit with an examiner. Language production was measured using a self-report measure of pragmatic language and a verbal fluency task (i.e., semantic and phonemic), and speech was measured with diadochokinetic (DDK) articulatory rate (per second). We assessed working memory (i.e., Digit Span) and verbal inhibition (Hayling Sentence Completion). Participants self-reported symptoms of tremor using the Tremor Disability Questionnaire. Statistical analysis included ANCOVAs and Pearson partial correlations controlling for age and education.

**Results**: Significant differences from controls were observed in DDK rate (*F* = 5.94, *p* = 0.018) for females and semantic fluency for males (*F* = 5.84, *p* = 0.025). For females with the premutation, working memory was associated with DDK rate (*r* = − 0.63, *p* < 0.001) and pragmatics (*r* = − 0.40, *p* = 0.026); these patterns were not seen in female controls. For males with the premutation, verbal inhibition was associated with phonemic fluency (*r* = 0.73, *p* = 0.042) and pragmatics (*r* = − 0.70, *p* = 0.036); these patterns were not seen in male controls. Tremor was not associated with speech or language in either group.

**Conclusion**: Groups differed in motor speech coordination (females) and language (males) compared to respective controls, with links to executive functioning. In concert with recent evidence that executive symptoms may reflect a prodrome of FXTAS, this could reflect co-occurring speech and language differences prior to onset.

**Grants**: National Institute on Deafness and Other Communication Disorders R21DC020257 (PI: Maltman) and the University of Arizona.

## P33 “Explore your potentials”: a Therapeutic Education Program for adolescents with intellectual disability

### Christelle Rougeot-Jung, Pauline Moreau-Dialinas, Emilie Decrette, Emeline Peyric, Odette Venancio, Nadine Roget, Amélie Clement, Vincent Des Portes

#### Le CRDI de Lyon (Centre de référence des déficiences intellectuelles de causes rares), Hôpital Femme Mère Enfant, Lyon, France

*Orphanet Journal of Rare Diseases* 2025, **20(1)**: P33

**Background/objectives:** A therapeutic Education Program was set-up in March 2021 at the National Reference Centre for Fragile X and other intellectual disabilities in Lyon. The main objectives were to develop skills to deal with daily life; boost self-confidence; prevent challenging behaviors; and improve quality of life and autonomy.

**Methods:** Target Population: groups of 5–7 teenagers (12–18), boys and girls, affected with mild to moderate Intellectual Disability.

Organizers: a pluridisciplinary team including a program coordinator, a child neurologist, a child psychiatrist, 3 neuropsychologists and a nurse.

**Results and conclusions:** Thematic workshops: Four main topics are addressed during the sessions: (i) I know my strengths and weaknesses (2 sessions); I can manage my emotions (2 sessions); I'm growing up, my body is changing; and I understand my feelings of love. Two additional sessions are still in progress: I know my medications and the «administrative adventure» (module constructed with a social worker). A «café parents» was also initiated, with very interesting feedback from parents who participated  in this group.

Activity: 32 teenagers already attended the Program, with a personalized educational assessments at first visit, followed by a personalised 18-month course. 17 teenagers already achieved their course.

A 9 min video explains the genesis of the project and shows interviews of teenagers, professionals and parents who attended the Program.

